# Quercetin and Rutin as Modifiers of Aphid Probing Behavior

**DOI:** 10.3390/molecules26123622

**Published:** 2021-06-13

**Authors:** Katarzyna Stec, Bożena Kordan, Beata Gabryś

**Affiliations:** 1 Department of Botany and Ecology, University of Zielona Góra, Szafrana 1, 65-516 Zielona Góra, Poland; b.gabrys@wnb.uz.zgora.pl; 2 Department of Entomology, Phytopathology and Molecular Diagnostics, University of Warmia and Mazury in Olsztyn, Prawocheńskiego 17, 10-720 Olsztyn, Poland; bozena.kordan@uwm.edu.pl

**Keywords:** flavonoids, pea aphid, bird cherry-oat aphid, peach-potato aphid, stylet penetration, antifeedants

## Abstract

Rutin and its aglycone quercetin occur in the fruits, leaves, seeds, and grains of many plant species and are involved in plant herbivore interactions. We studied the effect of the exogenous application of rutin and quercetin on the probing behavior (= stylet penetration activities in plant tissues) of *Acyrthosiphon pisum* on *Pisum sativum, Myzus persicae* on *Brassica rapa* ssp. *pekinensis*, and *Rhopalosiphum padi* on *Avena sativa* using the electrical penetration graph technique (EPG = electropenetrography). The reaction of aphids to quercetin and rutin and the potency of the effect depended on aphid species, the flavonol, and flavonol concentration. Quercetin promoted probing activities of *A. pisum* within non-phloem and phloem tissues, which was demonstrated in the longer duration of probes and a trend toward longer duration of sap ingestion, respectively. *M. persicae* reached phloem in a shorter time on quercetin-treated *B. rapa* than on the control. Rutin caused a delay in reaching sieve elements by *A. pisum* and deterred probing activities of *M. persicae* within non-phloem tissues. Probing of *R. padi* was not affected by quercetin or rutin. The potency of behavioral effects increased as the applied concentrations of flavonols increased. The prospects of using quercetin and rutin in plant protection are discussed.

## 1. Introduction

Aphids (Hemiptera: Aphididae) are herbivorous insects with piercing-sucking mouthparts that ingest plant sap precisely from phloem vessels [1]. Such a way of feeding requires the insertion of the mouthparts’ stylets into plant tissues and their progressive movement toward sieve elements of the phloem. On their route to phloem, aphid stylets puncture cells of non-phloem tissues, mainly for gustatory purposes [2]. This behavioral pattern associated with host-plant selection and feeding explains why aphids became serious pests of agricultural and horticultural crops. Specifically, aphids injure plants directly by removing nutrients from the transporting vessels, the sieve elements, and indirectly by transferring viruses from infected to healthy plants [3]. The peach-potato aphid *Myzus persicae* (Hemiptera: Aphididae) alone is able to transmit more than 100 plant viruses among plants within over 40 families [4]. Interestingly, the indirect damage caused by aphids due to virus transmission exceeds their direct impact on crops [5]. Present-day strategies of aphid control are based mainly on neurotoxic insecticides, the use of which raises health and environmental issues [6,7,8]. At the same time, many aphid pest species have developed resistance to several classes of these insecticides [9,10]. As a consequence, alternative methods of aphid management are in high demand. One of the routes explored is the manipulation of aphid behavior so the insect is either disoriented or discouraged from feeding [11,12,13,14]. This is often achieved by the use of antifeedants or attractants, especially in the ‘push-pull’ strategies [15,16,17,18]. The most potent aphid behavior modifying chemicals come from natural sources and represent various groups of secondary plant compounds including flavonoids [11,12,19,20,21]. The synthesis of flavonoids in plants is also induced by herbivore attack including aphid infestation [21,22,23].

Rutin and its aglycone quercetin are well-known plant flavonoids that mediate insect–plant relationships and are parts of constitutive and induced plant resistance mechanisms [21,24,25,26]. However, each of these flavonols causes different effects on insect behavior depending on the insect and plant species involved. Exposure to quercetin increased the developmental time, the pre-reproductive period, and mortality, and decreased fecundity and the intrinsic rate of the natural increase of the pea aphid *Acyrthosiphon pisum* (Hemiptera: Aphididae) on an artificial diet [27], reduced the infestation of winter wheat (*Triticum aestivum*) (Poaceae) by nymphs and apterous females of the bird cherry-oat aphid *Rhopalosiphum padi* (Hemiptera: Aphididae) [28], and inhibited the immune system and affected the growth and development of silkworm *Bombyx mori* (Lepidoptera: Bombycidae) [29]. Quercetin concentration in plants increased in response to the mango aphid *Toxoptera odinae* (Hemiptera: Aphididae) infestation of Chinese tallow *Triadica sebifera* (Euphorbiaceae) [23]. High concentration of rutin was found in soybean (*Glycine max*) (Fabaceae) cultivars resistant to *Piezodorus guildinii* (Hemiptera: Pentatomidae) and *Anticarsia gemmatalis* (Lepidoptera: Noctuidae) [30,31,32] and in cassava (*Manihot esculenta*) (Euphorbiaceae) cultivars resistant to mealybug *Phenacoccus manihoti* (Hemiptera: Pseudococcidae) [24,33]. Infestation by the mealybug was followed by an increase in level of rutin [24,34]. Rutin is toxic to the woolly apple aphid *Eriosoma lanigerum* (Hemiptera: Pemphigidae) [35]. Increased mortality and decreased intensity of cockchafer *Melolontha melolontha* (Coleoptera: Scarabaeidae) feeding was observed on *Quercus robur* (Fagaceae) leaves sprayed with a solution of rutin but quercetin solution did not produce any behavioral or developmental effect [36]. At the same time, rutin is a phagostimulant to many polyphagous insects including the locust *Schistocerca americana* (Orthoptera: Acrididae) and caterpillars of the tobacco budworm *Heliothis virescens* (Lepidoptera: Noctuidae) [25]. 

The aim of our study was to assess the effect of exogenous application of rutin and its aglycone quercetin to the leaves of aphid host-plants on aphid behavior during probing (= aphid stylet penetration activities in plant tissues). We focused on three of the 15 aphid species of the most agricultural importance worldwide: the pea aphid, the major pest of leguminous plants; the bird cherry-oat aphid that attacks all major cereals and grasses; and the peach-potato aphid, an extremely polyphagous and highly efficient virus vector [4]. All aphid species under present study may encounter quercetin and rutin while probing in plant tissues under natural conditions [37]. Our motivation was that if any phase of aphid probing can be affected by topical application of quercetin and rutin to aphid host-plants, these flavonols will have potential for application in aphid control programs. To monitor aphid probing, we applied the electrical penetration graph technique known as EPG or electropenetrography. The great advantage of the EPG technique over any visual monitoring is the opportunity that it provides to pursue aphid immediate reactions to modifications in plant chemical composition [38,39,40].

## 2. Results

**Figure 1:**
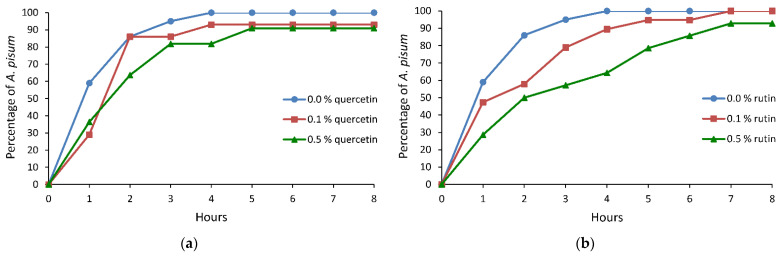
Cumulative proportion of *Acyrthosiphon pisum* individuals that reached phloem sieve elements on *Pisum sativum* treated with 0.0%, 0.1%, and 0.5% ethanolic solutions of (**a**) quercetin and (**b**) rutin.

**Figure 2:**
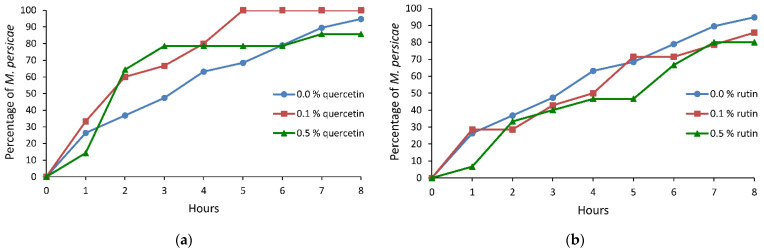
Cumulative proportion of *Myzus persicae* individuals that reached phloem sieve elements on *Brassica rapa* subsp. *pekinensis* treated with 0.0%, 0.1%, and 0.5% ethanolic solutions of (**a**) quercetin and (**b**) rutin.

**Figure 3:**
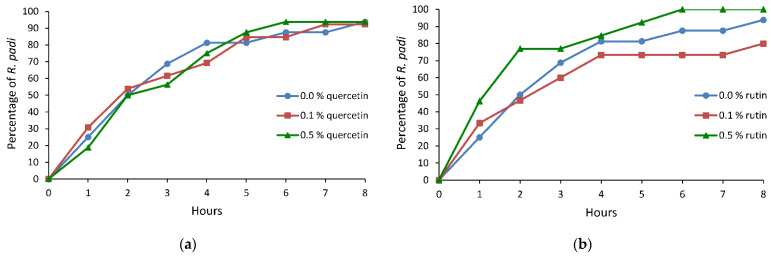
Cumulative proportion of *Rhopalosiphum padi* individuals that reached phloem sieve elements on *Avena sativa* treated with 0.0%, 0.1%, and 0.5% ethanolic solutions of (**a**) quercetin and (**b**) rutin.

The sequence of events during aphid stylet penetration in plant tissues was observed under semi-natural conditions, on plants treated topically with ethanolic solutions of quercetin and rutin. Electronic monitoring revealed two types of aphid behaviors irrespective of a treatment: (i) no-probing (= no-penetration), when aphid stylets remained outside plant tissues, and (ii) probing (= stylet penetration), when aphid stylets showed activities within plant tissues. Probing comprised non-phloem and phloem phases. The non-phloem phase consisted mainly of pathway and xylem stylet activities (waveforms C and G, respectively). Unidentified difficulties in penetration, classified also as derailed stylet mechanics (waveform F), also occurred, but only occasionally. The phloem phase consisted of watery salivation into sieve elements (waveform E1) and sap ingestion (waveform E2) (Table 1, Table 2 and Table 3, Figure 1, Figure 2 and Figure 3). 

### 2.1. Probing Behavior of Acyrthosiphon pisum on Pisum sativum

On peas treated with quercetin, the pea aphid activities associated with stylet penetration did not differ significantly in respect to the control, with an exception of the number and the duration of probes. Aphid probing was interrupted less frequently; specifically, the number of probes was 1.2 or 1.9 times lower and the probes were two times longer on leaves treated with 0.1% or 0.5% solution of quercetin, respectively, than on the control plants (Table 1). On average, the phloem vessels were reached within 1.4 h on both 0.1% and 0.5% quercetin-treated plants. Typically, the first contact with sieve elements included the periods of sap ingestion longer than 10 min (Table 1). More than 90% of aphids on 0.1% and 0.5% quercetin-treated peas reached phloem vessels within the first four hours of the experiment, but with a slight delay in comparison to control (Figure 1a). However, the total duration of phloem sap ingestion activity during the 8-h monitoring was similar in all aphids. Nevertheless, although statistical analysis did not reveal significant differences, there was a trend toward increasing the duration of the first sap ingestion period and the mean duration of individual sap ingestion periods on the quercetin-treated peas (Table 1).

On peas treated with rutin, statistically significant differences with respect to the control were detected only in the duration of time needed to attain the first sustained sap ingestion period, which was 1.8 or 2.5 times longer on 0.1% or 0.5% rutin-treated plants than on the control, respectively (Table 1). However, an observable trend toward decrease in the duration of the phloem phase and increase in the duration of pathway activity occurred, which translated into a lower value of the phloem phase index and a delay in finding phloem vessels on rutin-treated plants (Table 1). Within the first four hours of the experiment, 90% of aphids on 0.1% rutin-treated plants reached the phloem phase, while on 0.5% rutin-treated plants, 64% of aphids had contact with sieve elements within that period of time. On control plants, all aphids reached phloem vessels within four hours after access to plants (Figure 1b).

### 2.2. Probing and Settling Behavior of Myzus persicae on Brassica rapa subsp. pekinensis

On cabbage treated with quercetin, no statistically significant differences in the peach-potato aphid probing behavior were detected in relation to the control. However, there was a trend toward reduction of time needed to reach the first phloem phase and the first sustained sap ingestion period on quercetin-treated plants. The time to reach phloem vessels and reach sustained sap ingestion phase was 1.4 and 1.6 times shorter on 0.1% and 0.5% quercetin-treated plants than on the control (Table 2). Within the first four hours of the experiment, 80% of aphids reached phloem phase on 0.1% and 0.5% quercetin-treated plants while on the control it was – 63% (Figure 2a).

On cabbage treated with rutin, several aspects of aphid probing behavior differed significantly in respect to control. The total duration of no-probing was 1.3 or 2.8 times longer and time of no-probing before the first phloem phase was 1.7 or 3.3 times longer on 0.1% or 0.5% rutin-treated plants than on control, respectively. The number of probes was 1.2 or 1.5 times higher and the mean duration of probes was 2.0 or 3.0 times lower on 0.1% or 0.5% rutin-treated plants than on the control, respectively. In addition, there was a trend toward an increase of time needed to attain the first sustained sap ingestion period and a trend toward reduction in the duration of the first phloem phase and the mean duration of the sap ingestion periods (Table 2). Within the first four hours of the experiment, 50% of aphids reached the phloem phase on 0.1% or 0.5% rutin-treated plants (Figure 2b). 

### 2.3. Probing and Settling Behavior of Rhopalosiphum padi on Avena sativa

On oats treated with quercetin or rutin, no statistically significant alterations in the bird cherry-oat aphid probing behavior were recorded with respect to the control. However, there was a trend toward an increase in the duration of no-probing and a decrease in the total duration of the phloem phase as well as the duration of the first phloem phase on plants treated with both quercetin and rutin. Interestingly, while a similar proportion of aphids reached phloem sieve elements at a similar time on the quercetin-treated plants as on the control, on the 0.5% rutin-treated plants, more aphids reached sieve elements and did so much sooner than on the control and 0.1% rutin-treated plants (Table 3, Figure 3a,b). On the 0.5% rutin-treated plants, nearly 80% of aphids reached the phloem phase within two hours from the onset of the experiment. In the same period of time, 50% of aphids on the control and 0.1% rutin-treated plants had contact with phloem vessels (Figure 3b).

## 3. Discussion

In the present study, we demonstrated that neither quercetin nor rutin prevented aphids from probing in tissues of their host plants, irrespective of a treatment. Almost all aphids on all treated and untreated plants reached phloem vessels within eight hours of monitoring and were able to ingest sap for considerable periods of time without interruption. Nevertheless, each aphid species responded to exogenously applied flavonols but the responses were highly species-specific. Quercetin promoted probing activities of *A. pisum* on *P. sativum* within non-phloem and phloem tissues, which was demonstrated in the longer duration of probes and a trend toward longer duration of sap ingestion, respectively. Quercetin stimulated probing in non-phloem tissues also in *M. persicae* on *B. rapa. M. persicae* reached phloem in a shorter time on quercetin-treated plants than on the control. In *R. padi,* the addition of quercetin did not affect probing activities and the ability to reach phloem vessels in a significant way, but caused a slight reduction in phloem sap uptake, especially during the first contact with the phloem sap. In contrast, rutin caused a delay in reaching sieve elements and in the acceptance of phloem sap for sustained long-term feeding by *A. pisum*, which was probably due to the prolongation of time spent on probing in non-phloem tissues. Within probing activities during the 8-h monitoring, the proportion of time devoted to sap ingestion was reduced in favor of pathway activities on rutin-treated *P. sativum*. Rutin deterred significantly probing activities of *M. persicae* mainly within non-phloem tissues, which probably caused a lower success in reaching sieve elements by aphids on rutin-treated plants with respect to the control. In *R. padi,* the application of rutin did not produce a visible effect although a slight reduction in the duration of the first sap ingestion period occurred.

Generally, in all observed differences and trends in aphid probing, the potency of behavioral effects increased as the applied concentrations of quercetin and rutin increased. The most observable effect of an increase in flavonol concentration occurred in *M. persicae* on plants treated with rutin.

Insect activities associated with feeding can be affected at pre-ingestive (immediate effect associated with host finding and host selection processes involving gustatory receptors), ingestive (related to food transport and production, release, and digestion by salivary enzymes), and postingestive (long-term effects involving various aspects of digestion and absorption of food) phases [15]. Electropenetrography allows an insight into pre-ingestive and ingestive aphid behaviors [41]. The parameters describing aphid behavior during probing such as total time of probing, duration, and frequency of phloem sap ingestion events, number of probes, etc., are good indicators of plant suitability or interference of probing by chemical or physical factors in individual plant tissues [42,43]. Moreover, based on the characteristics of aphid stylet activities in different tissues, it is possible to predict aphid ability to acquire and inoculate non-persistent and persistent plant viruses [44]. At the level of non-phloem tissues, during brief intracellular probes in epidermis and parenchyma (mesophyll in leaves) that precede feeding in phloem vessels, small samples of plant sap are ingested for gustatory purposes as aphids lack external chemoreceptors and the taste organ is located in the hypopharynx [2,45,46]. During these brief probes, aphids may transmit non-persistent and semi-persistent viruses [47,48]. When aphid stylets reach sieve elements, persistent viruses may be transmitted [39,48]. The spread of viruses can be reduced by disrupting the feeding behavior of their aphid vectors [48]. 

Considering the results of the present experiments, it can be concluded that neither quercetin nor rutin is an effective blocker of aphid probing activities when applied as an ethanolic solution to plant surface. Such a method of application has been effective in modifying aphid stylet penetration activities in non-phloem and in phloem tissues by various groups of chemicals including flavonoids [49,50,51]. Here, we demonstrated that exogenously applied quercetin is either inactive behaviorally or weakly stimulatory for probing activities of *A. pisum, M. persicae*, and *R. padi* on their respective host-plants. Rutin, however, evoked significant negative responses in *A. pisum* and *M. persicae*, but not in *R. padi*. 

Quercetin and its derivatives including rutin occur ubiquitously in plants where they play crucial roles in plant cell metabolism [25,26,52,53,54,55]. It is possible that the three aphid species studied have adapted to tolerate a range of quercetin and rutin amounts in their diet under natural conditions. However, the levels of adaptation to quercetin and rutin seem to be different in different aphid species. Our results show that quercetin is better tolerated by *A. pisum, M. persicae*, and *R. padi* than rutin, and rutin is better tolerated by *A. pisum* and *R. padi* than *M. persicae*. Furthermore, the study by [27] showed that when added to artificial diets, only the high concentration of quercetin limited the diet uptake by *A. pisum* [27]. The concentration of quercetin applied in the present study stimulated probing but not sap ingestion by *A. pisum.*

Due to flavonoid nutritional importance, breeding attempts, conventional and involving genetic engineering, have been made to increase flavonoid levels in plants [54,56], which might influence different aspects of insect–plant interactions [26]. It is often expected that the elevated levels of secondary plant compounds in plant tissues may protect these plants against pathogens and herbivores [57]. The potential of quercetin to reduce herbivory has been reviewed extensively by [58]. Our studies show that the use of quercetin or rutin for the prevention of virus transmission by *A. pisum, M. persicae*, and *R. padi* seems unlikely. Aphid probing activities that are crucial for the transmission of non-persistent and persistent viruses are not affected significantly by these flavonols. Nevertheless, as far as the limitation of direct damage due to aphid infestation is concerned, the application of rutin can be considered against *M. persicae*. Rutin shows potential to discourage the peach-potato aphid from probing in plant tissues. 

## 4. Materials and Methods

### 4.1. Cultures of Plants and Aphids

Laboratory clones of *Acyrthosiphon pisum*, *Myzus persicae,* and *Rhopalosiphum padi* were maintained on *Pisum sativum* cv. Milwa, *Brassica rapa* ssp. *pekinensis* cv. Hilton, and *Avena sativa* cv. Komfort, respectively, in the laboratory at 20 °C, 65% r.h., and L16:8D photoperiod. Aphid clones have been maintained in the laboratory of Department of Botany and Ecology, University of Zielona Góra, Poland for at least 10 years. One- to seven-day old apterous aphid females and 3-week old plants were used for the experiments. Plants used for experiments were the same plant species and cultivars that were used for the rearing of aphids. All experiments were carried out under the same conditions of temperature, relative humidity, and photoperiod. The bioassays were started at 10–11 a.m.

### 4.2. Application of Quercetin and Rutin

Quercetin and rutin were purchased from Sigma–Aldrich (Poland). The flavonoids were dissolved in 70% ethanol to obtain 0.1% and 0.5% solutions. All compounds were applied on the adaxial and abaxial leaf surfaces by immersing one plant leaf of an intact plant in the ethanolic solution of a given compound for 30 seconds. [43]. Control leaves of similar size on the control intact plants were immersed in 70% ethanol that was used as a solvent for the studied compounds. Experiments were performed 1 h after the compounds’ application to allow for the evaporation of the solvent.

### 4.3. Behavioral Responses of Aphids

*Aphid Probing Behavior (No-Choice Test)*

Aphid probing (= aphid stylet penetration in plant tissues) was monitored using the electronic penetration graph technique (= electropenetrography) known as EPG, which is frequently employed in insect–plant relationship studies considering insects with sucking-piercing mouthparts [59]. In this experimental setup, aphids and plants are made parts of an electric circuit, which is completed when the aphid inserts its stylets into the plant [60]. Weak voltage is supplied in the circuit, and all changing electric properties are recorded as EPG waveforms that can be correlated with aphid activities and stylet position in plant tissues [1,47]. In the present study, aphids were attached to a golden wire electrode with conductive silver paint and starved for 1 h prior to the experiment. Probing behavior of 20 apterous females per studied flavonoid/aphid combination was monitored for 8 h continuously with four-channel DC EPG recording equipment. Each aphid was given access to a freshly prepared plant leaf. Each plant–aphid set was considered as a replication and was tested only once. The number of replications (= EPG recordings) for each plant cultivar was 20. Recordings that terminated due to aphid falling from the plant or where EPG signal was unclear were discarded from analysis. Only the replications that included complete 8 h recordings were kept for analysis. All experiments were carried out under the same conditions of temperature, relative humidity (r.h.), and photoperiod as those used for the rearing of plants and aphids. All bioassays started at 10:00–11:00 h MEST (Middle European Summer Time).

Signals were saved on the computer and analyzed using the PROBE 3.1 software provided by W.F. Tjallingii (www.epgsystems.eu; Wageningen 6703 CJ, The Netherlands) The following aphid behaviors were distinguished: no penetration (waveform ‘np’ – aphid stylets outside the plant), pathway phase-penetration of non-phloem tissues (waveforms ‘ABC’), derailed stylet movements (waveform ‘F’), salivation into sieve elements (waveform ‘E1′), ingestion of phloem sap (waveform ‘E2′), and ingestion of xylem sap (waveform ‘G’). The E1/E2 transition pattern was split in two between E1 and E2. ‘G’ and ‘F’ occurred sporadically; therefore these events were combined with pathway activities in all calculations and defined as non-phloem activities. The waveform patterns that were not terminated before the end of the experimental period (8 h) were included in the calculations. The parameters derived from EPGs were analyzed according to their frequency and duration in a configuration related to activities in peripheral and vascular tissues. In non-sequential parameters, when a given waveform had not been recorded for an individual, the duration of that waveform was given the value of 0. In sequential parameters, when parameters related to phloem phase (E1 or E2) were involved, only aphids that reached phloem phase were included in the statistical analysis. 

### 4.4. Statistical Analysis

EPG parameters describing aphid probing behavior (no-choice test) were calculated manually and individually for every aphid and the mean and standard errors were subsequently calculated using the EPG analysis Excel worksheet created for this study. The parameters derived from EPGs were analyzed according to their frequency and duration in a configuration related to activities in peripheral and vascular tissues. The results were statistically analyzed using the Kruskal–Wallis non-parametric test (Statistica 13.3 package).

## Figures and Tables

**Table 1:** EPG-recorded stylet penetration activities of *Acyrthosiphon pisum* on *Pisum sativum* treated with 0.1% and 0.5% ethanolic solutions of quercetin and rutin.

EPG Variable ^1^	Control	Quercetin		Rutin	
		0.1%	0.5%	0.1%	0.5%
*General aspects*	n = 22	n = 14	n = 11	n = 19	n = 14
Total duration of no-probing (h) ^2^	0.4 ± 0.1 ^a,A^	0.4 ± 0.2 ^a^	0.3 ± 0.1 ^a^	0.4 ± 0.1 ^A^	0.6 ± 0.1 ^A^
Total duration of pathway C + F + G (h) ^2^	2.9 ± 0.3 ^a,A^	3.2 ± 0.5 ^a^	3.2 ± 0.6 ^a^	4.6 ± 0.3 ^A^	4.7 ± 0.4 ^A^
Total duration of phloem phase E1 + E2 (h) ^2^	4.7 ± 0.3 ^a,A^	4.4 ± 0.6 ^a^	4.5 ± 0.6 ^a^	2.9 ± 0.4 ^A^	2.7 ± 0.4 ^A^
Phloem phase index (E1 + E2) / (C + E1 + E2 + G + F) ^2^	0.62 ± 0.04 ^a,A^	0.56 ± 0.08 ^a^	0.58 ± 0.08 ^a^	0.38 ± 0.05 ^A^	0.37 ± 0.05 ^A^
Number of probes ^2^	15.5 ± 1.9 ^a,A^	12.7 ± 2.4 ^a,b^	8.1 ± 2.6 ^b^	14.6 ± 1.7 ^A^	15.9 ± 1.7 ^A^
Mean duration of a probe (h) ^2^	0.8 ± 0.2 ^a,A^	1.6 ± 0.6 ^a,b^	1.7 ± 0.3 ^b^	0.8 ± 0.2 ^A^	0.6 ± 0.1 ^A^
*Probing in non-phloem tissues* *before first phloem phase*	n = 22	n = 13	n = 10	n = 19	n = 13
Number of probes before first phloem phase ^3^	5.0 ± 1.0 ^a,A^	6.0 ± 1.4 ^a^	4.0 ± 2.0 ^a^	5.4 ± 1.2 ^A^	6.2 ± 1.5 ^A^
Duration of first probe (min) ^3^	31.9 ± 12.7 ^a,A^	45.9 ± 36.2 ^a^	37.6 ± 22.4 ^a^	26.4 ± 12.1 ^A^	23.2 ± 11.4 ^A^
Time from first probe to first phloem phase (h) ^3^	1.1 ± 0.2 ^a,A^	1.4 ± 0.2 ^a^	1.4 ± 0.4 ^a^	1.9 ± 0.4 ^A^	2.3 ± 0.6 ^A^
Time from first probe to first sustained sap ingestion phase E2 > 10 min (h) ^4^	1.1 ± 0.2 ^a,A^	1.4 ± 0.2 ^a^	1.8 ± 0.4 ^a^	2.0 ± 0.4 ^A,B^	2.7 ± 0.5 ^B^
*Probing in phloem tissues*	n = 22	n = 13	n = 10	n = 19	n = 13
Duration of first phloem phase E1 + E2 (h) ^3^	1.5 ± 0.4 ^a,A^	2.8 ± 0.2 ^a^	2.9 ± 0.8 ^a^	1.4 ± 0.3 ^A^	1.1 ± 0.2 ^A^
Mean duration of phloem sap ingestion phase E2 (h) ^3^	1.4 ± 0.4 ^a,A^	1.9 ± 0.6 ^a^	2.0 ± 0.4 ^a^	0.8 ± 0.1 ^A^	1.0 ± 0.2 ^A^
Phloem salivation index E1 / (E1 + E2) ^3^	0.04 ± 0.01 ^a,A^	0.02 ± 0.01 ^a^	0.02 ± 0.01 ^a^	0.07 ± 0.04 ^A^	0.04 ± 0.01 ^A^

^1^ C = pathway, F = unidentified difficulties in penetration, G = xylem sap ingestion, E1 = watery salivation into sieve elements, E2 = phloem sap ingestion, np = no-probing; ^2^ All replicates (= individual EPG recordings) were included in statistical analysis irrespective of the presence of phloem phase; ^3^ Only replicates that embraced at least phloem phase E1 were included in the statistical analysis; ^4^ Only replicates that embraced phloem sap ingestion phase E2 > 10 min were included in the statistical analysis; n = number of replicates included in statistical analysis. Values represent means ± SD. Different letters in rows denote statistically significant differences: small letters refer to the comparison among aphids on control, 0.1% and 0.5% quercetin-treated leaves and capital letters refer to the comparison among aphids on control, 0.1% and 0.5% rutin-treated leaves (Kruskal–Wallis test, *p* < 0.05).

**Table 2:** EPG-recorded stylet penetration activities of *Myzus persicae* on *Brassica rapa* subsp. *pekinensis* treated with 0.1% and 0.5% ethanolic solutions of quercetin and rutin.

EPG Variable ^1^	Control	Quercetin		Rutin	
		0.1%	0.5%	0.1%	0.5%
*General aspects*	n = 19	n = 15	n = 14	n = 14	n = 15
Total duration of no-probing (h) ^2^	0.9 ± 0.2 ^a,A^	1.1 ± 0.3 ^a^	1.2 ± 0.4 ^a^	1.2 ± 0.2 ^A,B^	2.5 ± 0.4 ^B^
Total duration of pathway C + F + G (h) ^2^	3.7 ± 0.4 ^a,A^	2.9 ± 0.3 ^a^	3.9 ± 0.5 ^a^	4.0 ± 0.5 ^A^	3.6 ± 0.4 ^A^
Total duration of phloem phase E1 + E2 (h) ^2^	3.4 ± 0.5 ^a,A^	4.0 ± 0.5 ^a^	2.8 ± 0.7 ^a^	2.8 ± 0.6 ^a^	1.8 ± 0.6 ^A^
Phloem phase index ^2^ (E1 + E2) / (C + E1 + E2 + G + F)	0.46 ± 0.07 ^a,A^	0.56 ± 0.06 ^a^	0.37 ± 0.09 ^a^	0.39 ± 0.08 ^A^	0.28 ± 0.08 ^A^
Number of probes ^2^	27.1 ± 4.6 ^a,A^	27.5 ± 4.0 ^a^	26.4 ± 3.5 ^a^	32.4 ± 5.3 ^A,B^	43.9 ± 5.3 ^B^
Mean duration of a probe (h) ^2^	0.6 ± 0.2 ^a,A^	0.5 ± 0.1 ^a^	0.4 ± 0.1 ^a^	0.3 ± 0.1 ^A,B^	0.2 ± 0.1 ^B^
*Probing in non-phloem tissues* *before first phloem phase*					
Number of probes before first phloem phase ^3^	13.2 ± 2.3 ^a,A^ n = 18	14.2 ± 3.1 ^a^ n = 15	10.7 ± 2.5 ^a^ n = 12	19.1 ± 4.1 ^A,B^ n = 12	31.3 ± 6.0 ^B^ n = 12
Duration of first probe (min) ^3^	21.2 ± 14.8 ^a,A^ n = 18	1.0 ± 0.2 ^a^ n = 15	22.8 ± 19.4 ^a^ n = 12	1.5 ± 0.5 ^A,B^ n = 12	0.4 ± 0.1 ^B^ n = 12
Time from first probe to first phloem phase (h) ^3^	3.0 ± 0.5 ^a,A^ n = 18	2.1 ± 0.4 ^a^ n = 15	1.9 ± 0.4 ^a^ n = 12	3.1 ± 0.7 ^A^ n = 12	3.3 ± 0.6 ^A^ n = 12
Time from first probe to first sustained sap ingestion phase E2 > 10 min (h) ^4^	3.3 ± 0.6 ^a,A^ n = 17	2.3 ± 0.4 ^a^ n = 15	2.1 ± 0.5 ^a^ n = 10	3.9 ± 0.8 ^A^ n = 12	4.0 ± 0.7 ^A^ n = 10
Total duration of no-probing before first phloem phase (h) ^3^	0.4 ± 0.1 ^a,A^ n = 18	0.5 ± 0.2 ^a^ n = 15	0.3 ± 0.1 ^a^ n = 12	0.7 ± 0.2 ^A,B^ n = 12	1.3 ± 0.2 ^B^ n = 12
*Probing in phloem tissues* ^2^	n = 18	n=15	n=12	n = 12	n = 12
Duration of first phloem phase E1 + E2 (h) ^3^	2.8 ± 0.6 ^a,A^	2.2 ± 0.6 ^a^	2.2 ± 0.7 ^a^	1.5 ± 0.5 ^A^	1.5 ± 0.7 ^A^
Mean duration of phloem sap ingestion phase E2 (h) ^3^	3.1 ± 0.5 ^a,A^	2.2 ± 0.6 ^a^	2.2 ± 0.7 ^a^	2.0 ± 0.5 ^A^	1.7 ± 0.7 ^A^
Phloem salivation index E1 / (E1 + E2) ^3^	0.08 ± 0.04 ^a,A^	0.03 ± 0.02 ^a^	0.04 ± 0.01 ^a^	0.02 ± 0.01 ^A^	0.07 ± 0.02 ^A^

^1^ C = pathway, F = unidentified difficulties in penetration, G = xylem sap ingestion, E1 = watery salivation into sieve elements, E2 = phloem sap ingestion, np = no-probing; ^2^ All replicates (= individual EPG recordings) were included in statistical analysis irrespective of the presence of phloem phase; ^3^ Only replicates that embraced at least phloem phase E1 were included in statistical analysis; ^4^ Only replicates that embraced phloem sap ingestion phase E2 > 10 min were included in statistical analysis; n = number of replicates included in statistical analysis. Values represent means ± SD. Different letters in rows denote statistically significant differences: small letters refer to the comparison among aphids on control, 0.1% and 0.5% quercetin-treated leaves, and capital letters refer to the comparison among aphids on the control, 0.1% and 0.5% rutin-treated leaves (Kruskal–Wallis test, *p* < 0.05).

**Table 3:** EPG-recorded stylet penetration activities of *Rhopalosiphum padi* on *Avena sativa* treated with 0.1% and 0.5% ethanolic solutions of quercetin and rutin.

EPG Variable ^1^	Control	Quercetin		Rutin	
		0.1%	0.5%	0.1%	0.5%
*General aspects*	n = 16	n = 13	n = 16	n = 15	n = 13
Total duration of no-probing (h) ^2^	0.6 ± 0.1 ^a,A^	1.0 ± 0.2 ^a^	1.0 ± 0.2 ^a^	0.9 ± 0.3 ^A^	0.9 ± 0.3 ^A^
Total duration of pathway C + F + G (h) ^2^	4.3 ± 0.5 ^a,A^	4.9 ± 0.4 ^a^	4.8 ± 0.4 ^a^	4.2 ± 0.5 ^A^	4.3 ± 0.6 ^A^
Total duration of phloem phase E1 + E2 (h) ^2^	3.1 ± 0.6 ^a,A^	2.1 ± 0.4 ^a^	2.2 ± 0.5 ^a^	2.9 ± 0.5 ^A^	2.7 ± 0.7 ^A^
Phloem phase index ^2^ (E1 + E2) / (C + E1 + E2 + G + F)	0.49 ± 0.07 ^a,A^	0.30 ± 0.06 ^a^	0.30 ± 0.06 ^a^	0.39 ± 0.07 ^A^	0.36 ± 0.09 ^A^
Number of probes ^2^	8.8 ± 1.6 ^a,A^	12.1 ± 1.7 ^a^	9.0 ± 1.4 ^a^	7.9 ± 1.3 ^A^	8.3 ± 1.5 ^A^
Mean duration of a probe (h) ^2^	1.3 ± 0.2 ^a,A^	0.7 ± 0.1 ^a^	1.6 ± 0.5 ^a^	1.7 ± 0.5 ^A^	1.6 ± 0.6 ^A^
*Probing in non-phloem tissues* *before first phloem phase*					
Number of probes before first phloem phase ^3^	2.4 ± 0.5 ^a,A^ n = 15	2.8 ± 0.8 ^a^ n = 12	2.5 ± 0.8 ^a^ n = 15	1.8 ± 0.4 ^A^ n = 12	2.5 ± 0.9 ^A^ n = 13
Duration of first probe (m) ^3^	69.4 ± 28.7 ^a,A^ n = 15	40.9 ± 9.3 ^a^ n = 12	89.2 ± 35.8 ^a^ n = 15	96.6 ± 45.0 ^A^ n = 12	104.0 ± 37.3 ^A^ n = 13
Time from first probe to first phloem phase (h) ^3^	1.6 ± 0.3 ^a,A^ n = 15	1.7 ± 0.4 ^a^ n = 12	2.3 ± 0.5 ^a^ n = 15	2.1 ± 0.6 ^A^ n = 12	1.6 ± 0.5 ^A^ n = 13
Time from first probe to first sustained sap ingestion phase E2 > 10 min (h) ^4^	2.9 ± 0.5 ^a,A^ n = 12	2.8 ± 0.7 ^a^ n = 10	4.1 ± 0.5 ^a^ n = 13	2.2 ± 0.3 ^A^ n = 11	2.8 ± 0.5 ^A^ n = 11
Total duration of no-probing before first phloem phase (h) ^3^	0.2 ± 0.04 ^a,A^ n = 15	0.2 ± 0.1 ^a^ n = 12	0.2 ± 0.1 ^a^ n = 15	0.3 ± 0.2 ^A^ n = 12	0.4 ± 0.2 ^A^ n = 13
*Probing in phloem tissues*	n = 15	n = 12	n = 15	n = 12	n = 13
Duration of first phloem phase E1 + E2 (h) ^3^	2.1 ± 0.7 ^a,A^	0.7 ± 0.3 ^a^	0.5 ± 0.2 ^a^	0.8 ± 0.4 ^A^	1.4 ± 0.8 ^A^
Mean duration of phloem sap ingestion phase E2 (h) ^3^	1.7 ± 0.6 ^a,A^	0.9 ± 0.3 ^a^	0.7 ± 0.2 ^a^	1.5 ± 0.4 ^A^	1.7 ± 0.7 ^A^
Phloem salivation index E1 / (E1 + E2) ^3^	0.17 ± 0.09 ^a,A^	0.07 ± 0.03 ^a^	0.12 ± 0.06 ^a^	0.09 ± 0.08 ^A^	0.08 ± 0.04 ^A^

^1^ C = pathway, F = unidentified difficulties in penetration, G = xylem sap ingestion, E1 = watery salivation into sieve elements, E2 = phloem sap ingestion, np = no-probing; ^2^ All replicates (= individual EPG recordings) were included in statistical analysis irrespective of the presence of phloem phase; ^3^ Only replicates that embraced at least phloem phase E1 were included in statistical analysis; ^4^ Only replicates that embraced phloem sap ingestion phase E2 > 10 min were included in statistical analysis; n = number of replicates included in statistical analysis. Values represent means ± SD. Different letters in rows denote statistically significant differences: small letters refer to the comparison among aphids on control, 0.1% and 0.5% quercetin-treated leaves and capital letters refer to the comparison among aphids on control, 0.1%, and 0.5% rutin-treated leaves (Kruskal–Wallis test, *p* < 0.05).

## Data Availability

Data are provided in the present article.
